# Maxillary labial frenectomy: a randomized, controlled comparative study of two blue (445 nm) and infrared (980 nm) diode lasers versus surgical scalpel

**DOI:** 10.1186/s12903-024-04364-w

**Published:** 2024-07-25

**Authors:** Farhad Sobouti, Aryousha Moallem Savasari, Mehdi Aryana, Neda Hakimiha, Sepideh Dadgar

**Affiliations:** 1 Dental Research Center, Mazandaran University of Medical Sciences, Sari, Iran; 2 Orthodontic Department, Faculty of Dentistry, Mazandaran University of Medical Sciences, Sari, Iran; 3 Faculty of Dentistry, Mazandaran University of Medical Sciences, Sari, Iran; 4 Student Research Committee, Faculty of Dentistry, Mazandaran University of Medical Sciences, Sari, Iran; 5 Laser Application in Medical Sciences Research Center, Shahid Beheshti University of Medical Sciences, Tehran, Iran

**Keywords:** Frenectomy, Diode lasers, Minor surgery, Pain, Wound healing, Oral surgery

## Abstract

**Background:**

This study aimed to compare the intra and postoperative complications of frenectomy procedure with a surgical scalpel versus 445 nm and 980 nm diode lasers.

**Methods:**

This randomized controlled clinical trial was conducted on 174 patients requiring maxillary labial frenectomy. After completion of fixed orthodontic treatment and primary closure of maxillary diastema, the patients were randomly assigned into three groups (*n* = 58): group 1 (frenectomy via 445 nm diode laser, continuous-wave, 1.5 W), group 2 (frenectomy via 980 nm laser, continuous-wave, 1.7 W), and control group (V-Y plasty technique via scalpel). Intra-operative bleeding, discomfort in chewing and speaking, pain, and tissue healing were compared among the groups immediately, at 7 and 30 days postoperatively using the Kruskal-Wallis, Mann-Whitney, and Chi-square tests.

**Results:**

Pain scores were significantly lower in group 1 compared to group 2 (immediately and day 7, *P* < 0.05). Significant faster tissue healing at days 7 and 30 were observed in group 1 compared to group 2 (*P* < 0.05). Group 1 was superior to the control group regarding lower intraoperative bleeding, discomfort in chewing and speaking (immediately and day 7), lower pain (immediately and day 7), and tissue healing (day 7) (*P* < 0.05 for all). Group 2 was significantly superior to the control group in lower intraoperative bleeding, discomfort in chewing and speaking (immediately and day 7), and better tissue healing (day 7) (*P* < 0.05 for all).

**Conclusions:**

In conclusion, diode laser frenectomy resulted in significantly lower intra and postoperative complications compared to the scalpel. Moreover, 445 nm diode laser showed significantly superior effects compared to 980 nm diode laser.

**Trial registration:**

The study protocol was registered on 29.10.2022 at the Iranian Registry of Clinical Trials (www.irct.ir) (registration number: IRCT20220630055326N1).

**Supplementary Information:**

The online version contains supplementary material available at 10.1186/s12903-024-04364-w.

## Background

A frenum is a thin fold of mucous membrane composed of muscle fibers and connective tissue. It connects the lip and buccal mucosa to the alveolar mucosa, gingiva, and underlying periosteum. High frenal attachments can lead to insufficient dental plaque removal and sulcular displacement due to the applied tension by frenal attachments [1]. In such cases, frenectomy should be necessarily performed. Frenectomy is also indicated in cases with diastema, gingival recession, labial movement limitation, and for some orthodontic and prosthodontic purposes [2, 3]. Relapse after completion of orthodontic treatment is one of the most challenging issues among orthodontists. It refers to the tendency of the teeth to return to their baseline position due to discontinuation of orthodontic forces [4]. Since a high-attached frenum is usually associated with midline diastema, frenectomy of the labial frenum is required to prevent relapse of maxillary midline diastema [5].

A frenectomy can be performed conventionally by a surgical scalpel, an electric scalpel (electro-surgery), or a laser (laser surgery) [6]. Laser technology relies on the utilization of monochromatic and coherent photons produced by the stimulated emission of radiation. The distinctive characteristics of laser light allow for its diverse applications in the field of medicine and surgery, particularly in the maxillofacial regions. Lasers can be employed for soft and hard tissue surgeries determined by the wavelength and irradiation parameters chosen. Examples of laser applications in maxillofacial surgery include incisional/excisional procedures, tissue ablation, and surgical hemostasis [7, 8]. Laser surgery has several advantages over conventional surgery, such as selective and precise interaction with the injured tissue, reduced scar tissue formation and tissue contraction, induction of complete hemostasis and subsequent improvement of the surgeon’s vision to the surgical field, and decreased need for suturing. Moreover, it reduces the risk of postoperative infection and the stress level of patients [9]. However, controversy exists regarding the superiority of frenectomy with laser or surgical scalpel. Some studies have reported higher levels of comfort, no or minimal postoperative pain, and superior mastication efficiency and speech following laser frenectomy [10]. Yadav et al. [11] reported lower bleeding and less need for analgesics in laser surgery compared to a scalpel. However, the clinical results were similar. Scalpel surgery is associated with surgical trauma, intraoperative bleeding, postoperative pain, and edema [1], while laser surgery has advantages such as less intraoperative bleeding, lower inflammation, and improved tissue healing. It also decreases the need for anesthetics and wound care, such as suturing and dressing, thus decreasing the patients’ fear of surgery [9].

A diode laser is a semi-conductive laser with high absorption in tissue chromophores such as melanin and particularly oxyhemoglobin, which makes it an available and safe modality for oral soft tissue procedures. Diode laser wavelengths between 800 and 980 nm are routinely used in dental practices [12, 13]. Recently, a novel 445 nm diode laser with blue light was introduced for surgical procedures [14]. This wavelength has maximum absorption in hemoglobin that enables it to fast soft tissue incision with minimal bleeding. It has been successfully used for many intraoral surgical procedures such as gingivectomy, impacted tooth exposure, and soft tissue biopsy with satisfactory results [15].

The comparison of diode lasers in the range of 800–900 nm to scalpel surgery has been previously documented [16, 17], but limited data are available on 445 nm blue laser [14]. Thus, this study aimed to compare the intra and postoperative complications following frenectomy using a surgical scalpel versus 445 nm and 980 nm diode lasers.

## Methods

This study was conducted at the Orthodontics Department of the School of Dentistry, Mazandaran University of Medical Sciences, during 15 months between 2021 and 2022. The study protocol was approved by the Ethics Committee of the university (IR.MAZUMS.REC.1401.338) and registered at the Iranian Registry of Clinical Trials (IRCT20220630055326N1).

### Trial design

A parallel-design randomized controlled clinical trial was carried out in which the experimental groups underwent maxillary frenectomy with 445 and 980 nm diode lasers, and the control group received conventional frenectomy with a surgical scalpel. The results were reported following the Consolidated Standards of Reporting Trials (CONSORT) guidelines [18].

### Participants, eligibility criteria, and settings

The inclusion criteria were (I) patients under fixed orthodontic treatment of the maxilla or both jaws, (II) a midline diastema between the maxillary central incisors due to the presence of maxillary high frenal attachments, and (III) the need for a frenectomy. The exclusion criteria were (I) patients under prosthodontic or periodontal treatment of the anterior maxilla and (II) patients with dental diastema without high frenal attachments.

The samples were selected among patients referred to two private orthodontic offices and an orthodontic clinic in Sari and Behshahr cities, Mazandaran, Iran, by convenience sampling.

### Interventions

A total of 174 patients were selected from those referred by their orthodontist for maxillary labial frenectomy following diastema closure prior to the bracket debonding process. Written informed consent was obtained from all participants prior to their enrollment. All patients had received fixed orthodontic treatment of the maxilla or both jaws. Three orthodontists performed the treatments with pre-adjusted 0.022’ 0.028-inch slot MBT brackets (American Orthodontics, Sheboygan, WI, USA), which lasted 15 to 24 months (mean: 17.1 months). The patients were then randomly assigned into three groups (*n* = 58) of 445 nm diode laser, 980 nm diode laser, and conventional scalpel surgery as the control group. First, infiltration anesthesia was induced by injection of 2% lidocaine plus 1:80,000 epinephrine (Darupakhsh, Tehran, Iran), similarly in all groups, then the procedure was conducted in each group as follows:

#### Control group (conventional scalpel surgery)

The frenum was held by a hemostat, and a V-shaped incision was made on its lower surface. The frenum was then displaced apically, and the V-shaped incision was converted to a Y-shaped incision, and sutured with 5 − 0 silk thread.

#### Experimental group 1 (445 nm diode laser)

Blue 445 nm diode laser (SiroLaser; Dentsply Sirona, Germany) with 1.5 W power in continuous-wave mode was used for a frenectomy. Upon initiation of irradiation, the 320-µm fiber tip was moved from the base towards the apex of the frenum in a non-contact mode without Suturing.

#### Experimental group 2 (980 nm diode laser)

Frenectomy was performed with a 980 nm diode laser (Doctor Smile, Italy) with 1.7 W power in continuous-wave mode. Upon initiation of irradiation, the 400-µm fiber tip was moved from the base towards the apex of the frenum with a brushing motion with no pressure and no suturing [5]. For safety purposes, both the patient and the operator wore protective glasses during laser irradiation in experimental groups.

Following frenectomy procedures in all groups, the patients received oral hygiene instructions and were instructed to use soft and cold food for the next 12 h [11]. Moreover, a 0.12% chlorhexidine gluconate mouth rinse (once a day for 1 min for five days) was prescribed [19]. Five hundred milligrams of acetaminophen (1–2 tablets) were also prescribed for pain relief if required, and the patients were asked to record the dosage and frequency of use.

#### Outcomes

Intraoperative bleeding, postoperative pain, discomfort in chewing and speaking, and tissue healing were the primary outcomes of the present study. There was no secondary outcome. To record the outcome measures, the following assessments were done immediately, on days 7 and 30 after surgery in an orthodontic office:

#### Bleeding

Intraoperative bleeding was scored and recorded by the surgeon using the following scoring system [20]:

- Score 0: No bleeding
- Score 1: Mild bleeding
- Score 2: Moderate bleeding
- Score 3: Severe bleeding

#### Pain and discomfort in chewing and speaking

Pain and discomfort in chewing and speaking were self-reported by patients immediately after surgery and at 7 and 30 days after treatment using a visual analog scale (VAS). Score 0 indicated minimal or no pain/discomfort, and 10 indicated maximum unbearable pain/discomfort [21].

#### Tissue healing

Tissue healing was assessed by a senior dental student immediately, at days 7 and 30 after surgery, using the following scoring system [22]:

- Score 1: Complete epithelialization
- Score 2: Incomplete epithelialization
- Score 3: Presence of ulcer
- Score 4: Tissue defect or necrosis

### Sample size calculation

The sample size was calculated to be 174 patients (*n* = 58 in each group) according to Sezgin et al. study [23], assuming the mean and standard deviation of periodontal healing parameters to be 0.93 ± 0.24 in group 1, 0.84 ± 0.13 in group 2, and 0.90 ± 0.14 in group 3 at 45 days after surgery, study power of 90%, and 95% confidence interval, using the formula for comparison of two means and G-Power software.

### Interim analyses and stopping guidelines

No interim analyses were performed, and no stopping guidelines were established.

### Randomization

The patients were randomly assigned into three groups by Random Allocation Software using 6-series blocks.

### Blinding

Blinding of patients, periodontist, and orthodontist was not possible in the present study. However, the examiner and the statistician who analyzed the data were blinded to the group allocation of patients and type of procedure.

### Statistical analysis

Data were analyzed by SPSS software (version 26 SPSS Inc., IL, USA). The normal distribution of data was evaluated by the Shapiro-Wilk and Kolmogorov-Smirnov tests, which showed a non-normal distribution of all data (*P* < 0.05). Thus, the groups were compared regarding quantitative variables (intraoperative bleeding, pain, discomfort in chewing and speaking, and tissue healing) by the Kruskal-Wallis (for general comparisons) and Mann-Whitney (for pairwise comparisons) tests. The Kruskal-Wallis test was also used to compare the trend of change in quantitative variables over time among the three groups. The three groups were compared regarding age by the Kruskal-Wallis test and gender by the Chi-square test. The level of statistical significance was set at 0.05.

## Results

### Participant flow

**Fig. 1:**
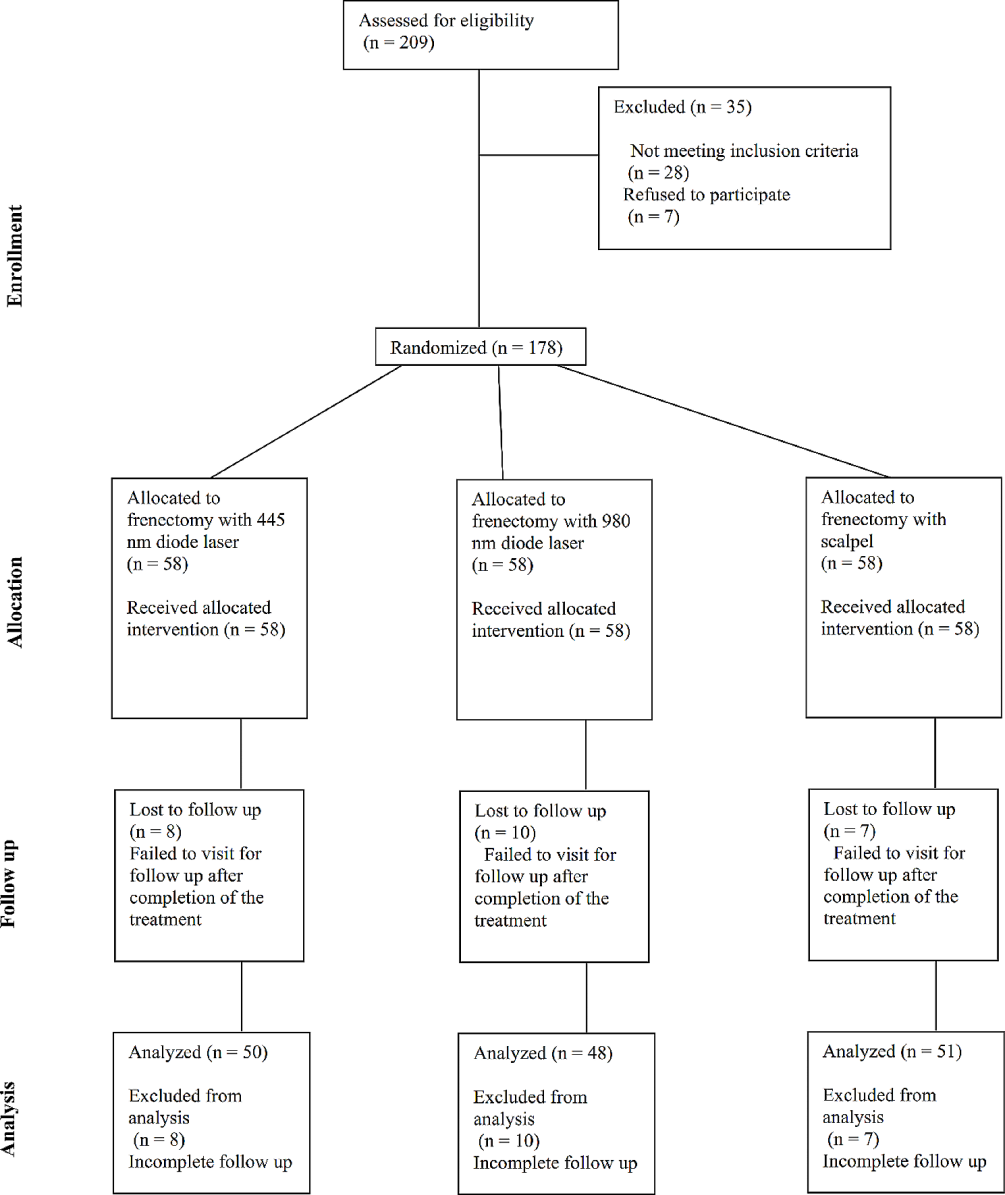
CONSORT flow-diagram of patient selection and allocation

The sample initially consisted of 174 patients, out of which 25 patients did not show up for the follow-ups and were excluded (Fig. 1). A total of 149 patients were eventually included, with a mean age of 18.6 years (female/ male: 92/57). Table 1 presents the demographic information of the participants in the study groups. There was no significant difference between groups regarding age (*P* = 0.381) or gender (*P* = 0.859).

**Table 1:** Demographic information of the participants in the three groups

Variable	Group	Scalpel surgery	445 nm diode	980 nm diode
Gender	Male	21	18	18
	Female	30	32	30
Mean age		17.8	19.2	16.8

### Subgroup analyses

Results for each outcome were measured and presented.

#### Intraoperative bleeding

**Fig. 2:**
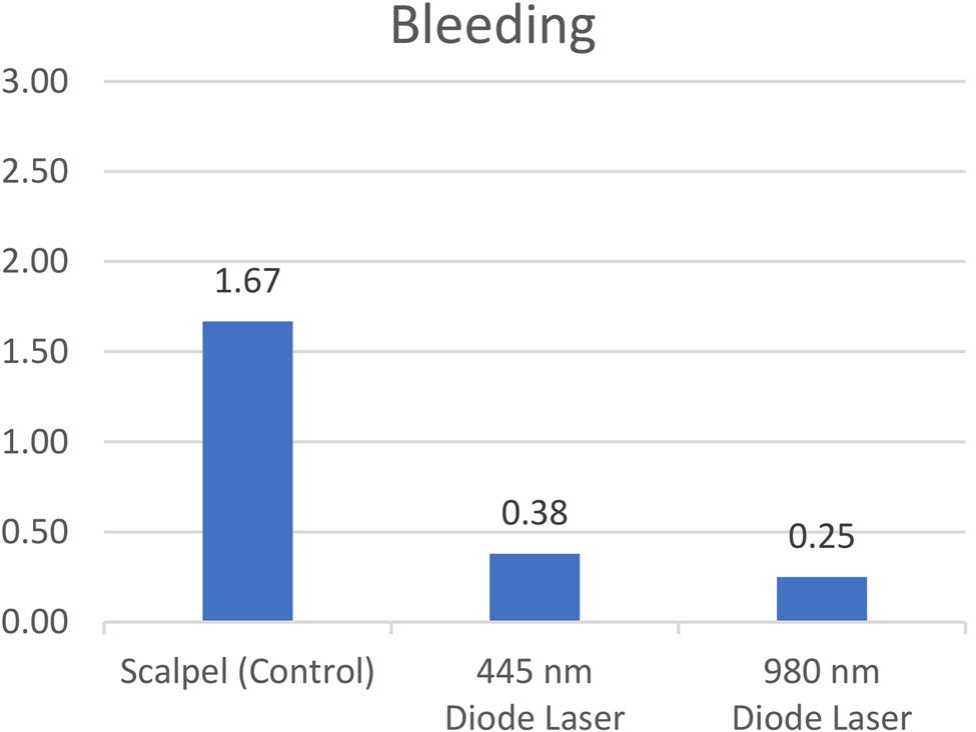
Mean score of intraoperative bleeding in the three groups (*p<0.05)

A significant difference was found among the study groups (*P* = 0.000). Pairwise comparisons of the groups showed significantly lower intraoperative bleeding in both laser groups compared to the scalpel group (*P* = 0.000). The difference between the two laser groups was not significant (*P* = 0.207) (Fig. 2).

#### Pain

**Fig. 3:**
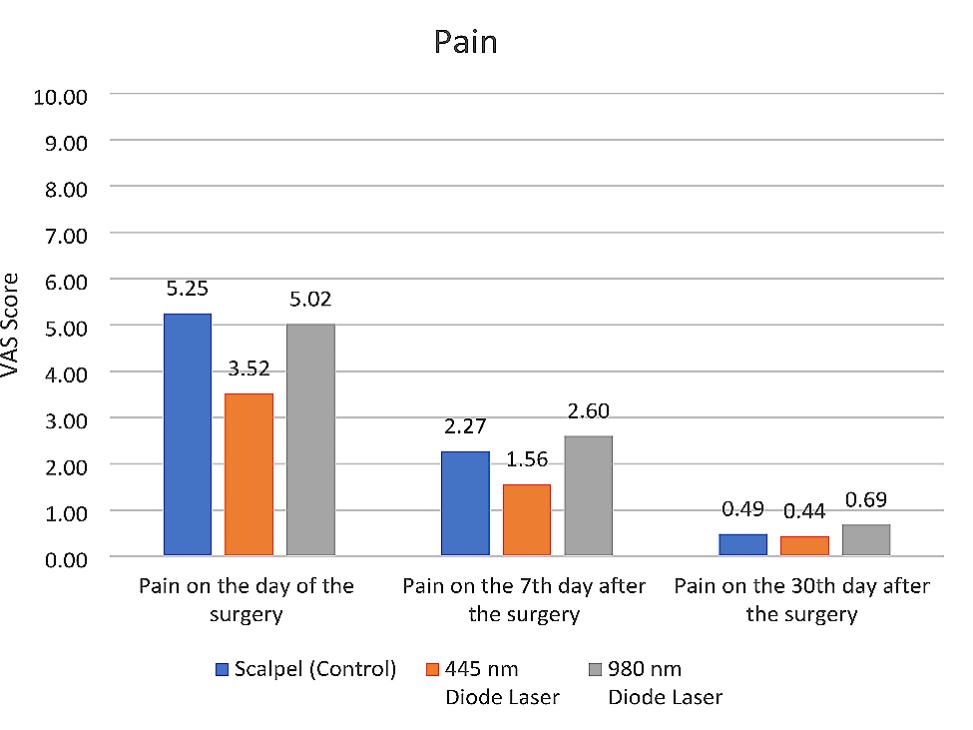
Mean score of postoperative pain in the three groups at different time points

A significant difference was found in pain score among the three groups immediately (*P* = 0.000) and at 7 days after surgery (*P* = 0.002), but not at 30 days postoperatively (*P* = 0.157). Pairwise comparisons revealed significantly lower pain scores immediately (*P* = 0.000) and postoperatively at 7 days (*P* = 0.009) in the 445 nm laser group than the scalpel group. The pain score was significantly lower in the 445 nm laser group than the 980 nm laser group immediately after surgery (*P* = 0.000) and at 7 days (*P* = 0.001). No other significant differences were noted at any time point (*P* > 0.05) (Fig. 3).

#### Analgesic intake

No analgesic intake was reported by patients in 445 nm and 980 nm diode laser groups. Four patients reported analgesic intake in the scalpel surgery group.

#### Discomfort in chewing and speaking

**Fig. 4:**
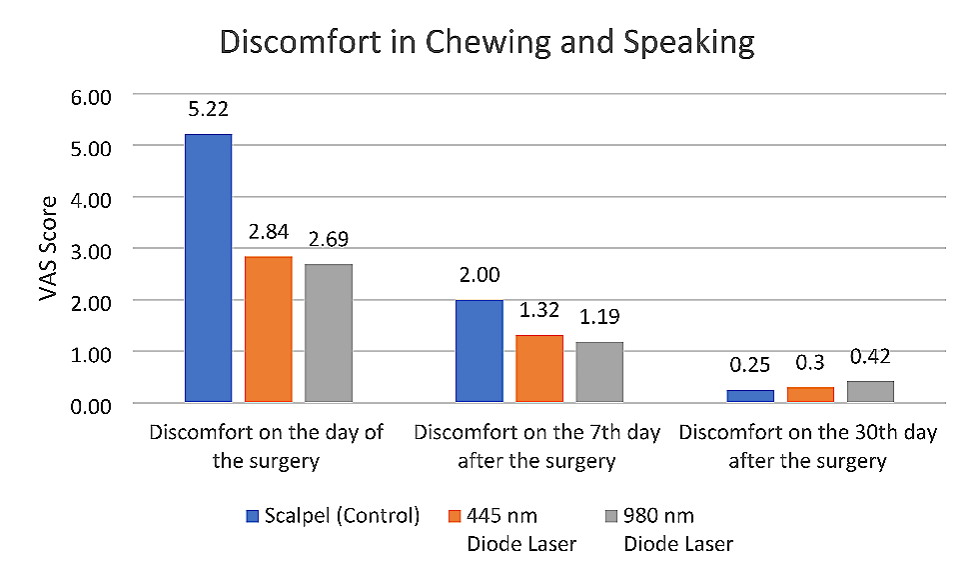
Mean score of discomfort in chewing and speaking in the three groups at different time points

The three groups had a significant difference in this regard immediately (*P* = 0.000) and at 7 days (*P* = 0.007) after surgery, but not at 30 days postoperatively (*P* = 0.249). Pairwise comparisons revealed significantly lower discomfort in chewing and speaking immediately (*P* = 0.000) and at 7 days (*P* = 0.012) after surgery in 445 nm diode laser group than the scalpel group. Also, such problems were significantly lower in 980 nm laser group than the scalpel group immediately (*P* = 0.000) and at 7 days (*P* = 0.004) after surgery. The difference between the two laser groups was not significant at any time point (*P* > 0.05) (Fig. 4).

#### Tissue healing

**Fig. 5:**
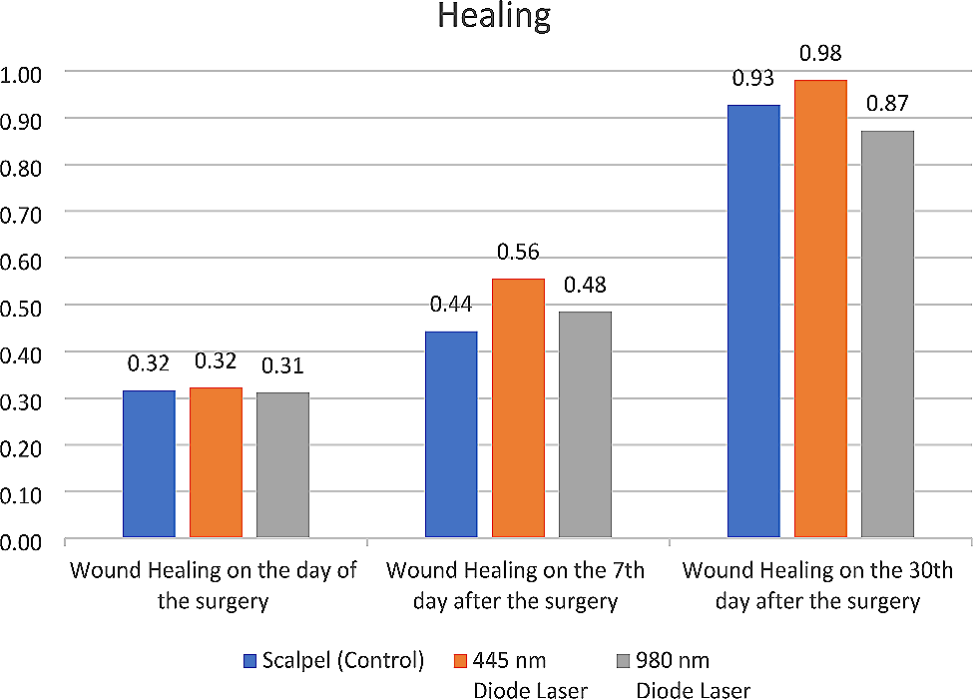
Mean score of tissue healing in the three groups at different time points

**Fig. 6:**
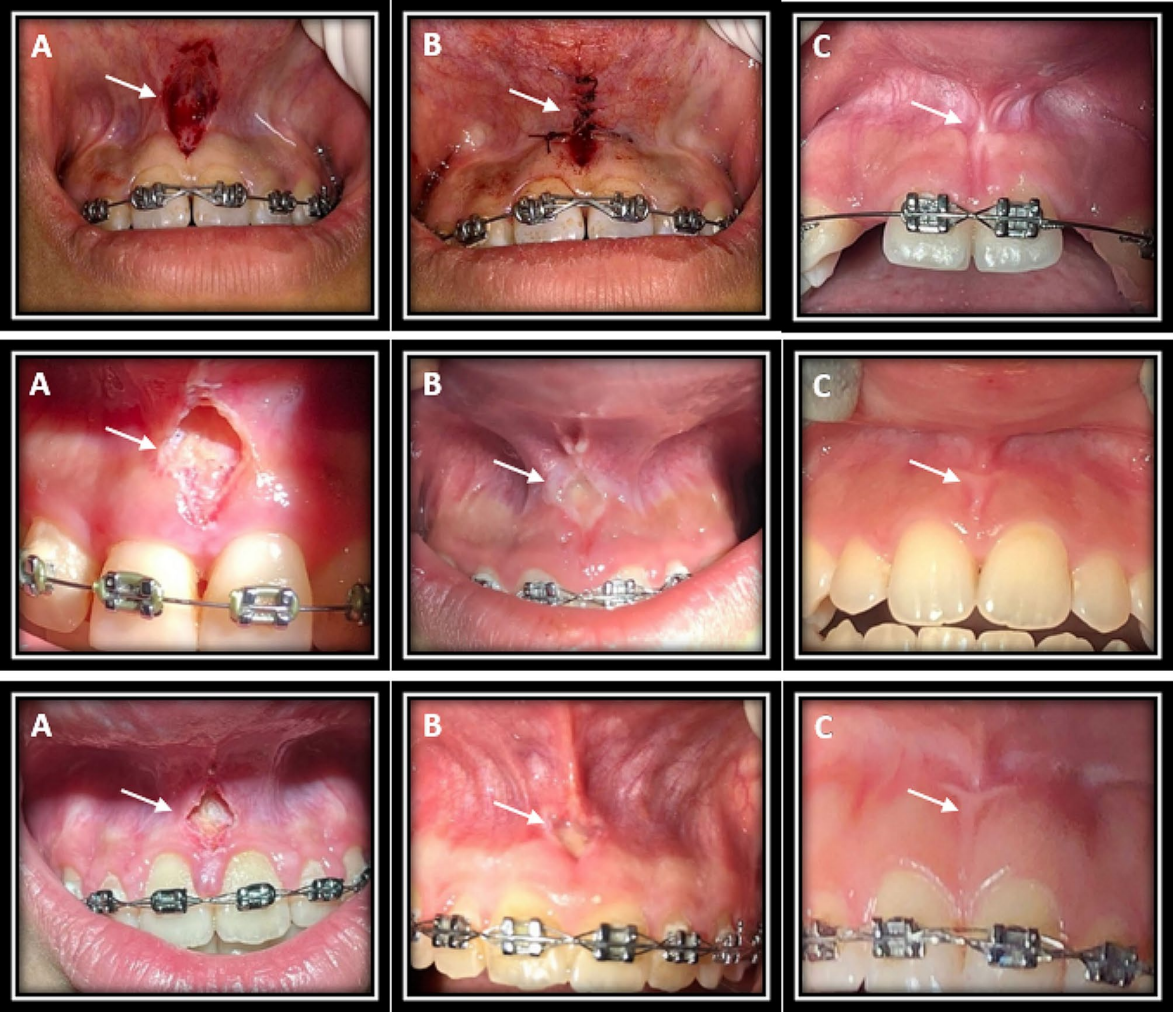
Intraoral photographs of tissue healing after frenectomy by a surgical scalpel (upper row), 445 nm diode laser (middle row), and 980 nm diode laser (lower row) immediately after surgery (**A**), 7 days after surgery (**B**), and 30 days after surgery (**C**)

Tissue healing was significantly different among the three groups at 7 days postoperatively (*P* = 0.000), but not immediately (*P* = 0.334) or at day 30 (*P* = 0.074). Pairwise comparisons showed significantly faster tissue healing at 7 days in 445 nm laser group than the scalpel group (*P* = 0.000). Also, tissue healing was significantly higher in 980 nm laser group than the scalpel group at 7 days (*P* = 0.038). Tissue healing in 445 nm laser group was faster than that in 980 nm laser group at 7 (*P* = 0.003) and 30 (*P* = 0.024) days. No other significant differences were noted at any time points (*P* > 0.05) (Fig. 5). Figure 6 shows intraoral photographs of tissue healing over time among three groups.

All of the pairwise and total differences between the three groups are shown in supplementary file S1.

### Comparison of the trend of change in quantitative variables over time among the three groups

The three groups were significantly different in the trend of reduction in discomfort in chewing and speaking from the time of surgery to day 7 (*P* = 0.000) and from the time of surgery to day 30 (*P* = 0.000). The three groups had a significant difference in the trend of change in pain score between the time of surgery and 30 days (*P* = 0.000, and tissue healing between day 1 and day 7 (*P* = 0.005). No other significant differences were found in this respect (*P* > 0.05).

Pairwise comparisons of the groups regarding the reduction of pain and discomfort in chewing and speaking from the time of surgery to 7 and 30 days and tissue healing from day 1 to days 7 and 30 revealed significant differences between 445 nm diode laser and scalpel group in favor of 445 nm laser group in all variables at all the tested time intervals (*P* < 0.05) except for tissue healing from day 1 to day 30, which was not significantly different between the abovementioned two groups (*P* > 0.05). A pairwise comparison of 980 nm laser and scalpel group only revealed a significant difference in the reduction of discomfort in chewing and speaking from the time of surgery to day 7 (*P* = 0.000, and day 30 (*P* = 0.000) in favor of 980 nm laser group. Also, a significant difference between the two laser groups was only found in the trend of reduction of pain from the time of surgery to 30 months (*P* = 0.000) in favor of 445 nm laser group. No other significant differences were noted (*P* > 0.05).

### Pairwise comparison of scalpel surgery with diode laser groups

Table 2 presents the mean and mean rank of quantitative variables in the scalpel surgery and diode laser groups. Significant differences were noted between the scalpel surgery and diode laser groups in intraoperative bleeding, discomfort in chewing and speaking, and pain immediately and 7 days postoperatively in favor of diode laser groups (*P* < 0.05).

**Table 2:** Mean and mean rank of quantitative variables in the scalpel surgery and diode laser groups

Quantitative variable	Group	Number	Mean	Std. deviation	Mean rank	Sum of ranks
Intraoperative bleeding	Scalpel surgery	51	1.67	0.712	115.67	5899.00
	Diode lasers	98	0.32	0.636	53.84	5276.00
Discomfort in chewing and speaking immediately after surgery	Scalpel surgery	51	5.22	1.724	106.29	5421.00
	Diode lasers	98	2.77	1.936	58.71	5754.00
Discomfort in chewing and speaking after 7 days	Scalpel surgery	51	2.00	1.483	89.87	4583.50
	Diode lasers	98	1.26	1.326	67.26	6591.50
Discomfort in chewing and speaking after 30 days	Scalpel surgery	51	0.25	0.595	70.23	3581.50
	Diode lasers	98	0.36	0.646	77.48	7593.50
Pain score immediately after surgery	Scalpel surgery	51	5.25	1.742	90.30	4605.50
	Diode lasers	98	4.26	1.658	67.04	6569.50
Pain score after 7 days	Scalpel surgery	51	2.27	1.297	79.39	4049.00
	Diode lasers	98	2.07	1.528	72.71	7126.00
Pain score after 30 days	Scalpel surgery	51	0.49	0.674	74.02	3775.00
	Diode lasers	98	0.56	0.787	75.51	7400.00
Tissue healing after 1 day	Scalpel surgery	51	3.16	0.367	75.19	3834.50
	Diode lasers	98	3.15	0.362	74.90	7340.50
Tissue healing after 7 days	Scalpel surgery	51	2.25	0.560	89.13	4545.50
	Diode lasers	98	1.93	0.437	67.65	6629.50
Tissue healing after 30 days	Scalpel surgery	51	1.08	0.272	74.84	3817.00
	Diode lasers	98	1.08	0.275	75.08	6358.00

### Comparison of the trend of change in quantitative variables over time between the scalpel surgery and diode laser groups

Table 3 compares the trend of reduction in pain and discomfort in chewing and speaking from the day of surgery to 7 and 30 days, and also tissue healing from day 1 to days 7 and 30 between the scalpel surgery and diode laser groups. As shown, significant differences were noted in all the variables in favor of the laser groups (*P* < 0.05), except for tissue healing between day 1 and day 30, which was not significantly different (*P* > 0.05).

**Table 3:** Comparison of the trend of reduction in pain and discomfort in chewing and speaking from the day of surgery to 7 and 30 days, and also tissue healing from day 1 to days 7 and 30 between the scalpel surgery and diode laser groups

Quantitative variable	Group	Number	Mean	Std. deviation	Mean rank	Sum of ranks
Discomfort in chewing and speaking days 0–7	Scalpel surgery	51	-3.2157	1.74715	50.72	2586.50
	Diode lasers	98	-1.5102	1.84024	87.64	8588.50
Discomfort in chewing and speaking days 0–30	Scalpel surgery	51	-4.9608	1.59951	41.34	2108.50
	Diode lasers	98	-2.4082	1.84356	92.52	9066.50
Pain score days 0–7	Scalpel surgery	51	-2.9804	2.07355	64.23	3275.50
	Diode lasers	98	-2.1837	1.68894	80.61	7899.50
Pain score days 0–30	Scalpel surgery	51	-4.7647	1.81756	58.56	2986.50
	Diode lasers	98	-3.6939	1.68320	83.56	8188.50
Tissue healing days 1–7	Scalpel surgery	51	-0.9020	0.67097	87.59	4467.00
	Diode lasers	98	-1.2245	0.50829	68.45	6708.00
Tissue healing days 1–30	Scalpel surgery	51	-2.0784	0.48345	74.62	3805.50
	Diode lasers	98	-2.0625	0.56139	75.20	7369.50

## Discussion

This study compared intraoperative bleeding, postoperative complications, and healing after a frenectomy procedure by a surgical scalpel versus 445 nm and 980 nm diode lasers. The results showed significantly lower intraoperative bleeding in laser groups compared to the scalpel group, with no significant differences between the laser groups. Gobbo et al. [24] reported similar results regarding the comparison of 445 nm and 980 nm lasers for excision of benign oral lesions. Similarly, no significant differences regarding bleeding scores between 445 nm and 940 nm diode lasers in gingival depigmentation were shown [20]. The superiority of 940 nm laser-mediated gingivectomy to a scalpel in terms of lower intraoperative bleeding was reported by Elif [25] and Sobouti et al. [26]. The optimal efficacy of diode laser in the reduction or elimination of intraoperative bleeding has been confirmed in previous studies [5, 27]. This hemostatic effect of laser gives the surgeon a much better view due to the laser-hemoglobin interaction [5].

Our findings revealed that the pain score in the laser groups was significantly lower than that in the surgical scalpel group. Immediately and at 7 days after surgery, the pain score in 445 nm laser group was significantly lower than that in the scalpel and 980 nm laser groups. This finding was consistent with several previous studies confirming lower pain scores in the laser group compared to scalpel surgery [20, 25, 26, 28]. Gobbo et al. [24] reported that 445 nm diode laser resulted in a lower pain score at 7 and 14 days compared to 970 nm laser. This discrepancy in the results may be attributed to using different anesthetic agents/techniques, types of surgery, and surgical sites [24].

In the present study, postoperative discomfort in chewing and speaking was significantly lower in laser groups than the scalpel surgery group immediately and at 7 days post-surgery. Similar results were reported in previous studies [22, 29]. Amaral et al. [30] compared 980 nm diode laser and scalpel surgery for removing fibrous hyperplasia and found no significant difference regarding discomfort in chewing and speaking between the two groups, which was in contrast to the present findings. This difference may be explained by the difference in surgical sites and type of surgery in the two studies. Also, patients with fibrous hyperplasia are usually denture wearers, which can explain the difference in the level of discomfort in chewing and speaking.

The current results showed that tissue healing accelerated in laser groups compared to the scalpel group at day 7, with superior results in 445 nm laser group. At 30 days postoperatively, tissue healing in the 445 nm laser group was significantly higher than in the 980 nm laser group. Noteworthy, we applied lasers in continues mode of irradiation. When comparing continuous and pulsed modes, it is observed that pulsed mode is linked with minimal thermal damage [31]. However, we ensured that the output power of the 445 and 980 nm lasers was maintained below 2 W (∼ 1.5 W), taking into account the device limitations and adhering to the safe settings validated by prior investigations [32]. Furthermore, the speed of ablation is enhanced through the use of a continuous setting [31]. The mechanism of action of laser is through enhancement of collagen synthesis and subsequent acceleration of healing of injured periodontal ligament [5]. This mechanism explains accelerated healing in laser groups [33]. The differences in tissue reactions observed between 445 nm and 980 nm diode lasers can be ascribed to the distinct optical characteristics of these wavelengths. Specifically, the 445 nm wavelength exhibits greater absorption in hemoglobin and melanin compared to other diode lasers, leading to enhanced cutting precision with reduced penetration depth and minimal thermal damages to underlying tissues [31, 33]. Reichelt et al. [34] compared wound healing following incision with 445 nm blue diode laser and 970 nm diode laser using a monolayer cell culture and reported faster tissue healing in the 445 nm laser group, consistent with our data. Palaia et al. [35], in their non-controlled clinical trial, performed 42 biopsies with 445 nm diode laser and reported normal tissue healing at 7 days post-surgery. They showed complete healing at 30 days after surgery, which was in agreement with the present findings. Qafmolla et al. [36] compared scalpel surgery and 980 nm diode laser for surgical removal of mucocele and reported faster tissue healing in the laser groups compared to the scalpel group at 4 weeks after surgery, which was in accordance with the present findings. Taher Agha and Polenik [20] found no significant difference in tissue healing between 445 nm and 940 nm diode lasers at 10 days after surgery, while a significant difference was noted between 445 and 980 nm diode lasers at 7 days in the present study. This controversy in the results of the two studies may be explained by the differences in the type of surgery, larger extent of the surgical field in depigmentation procedure, using different scales, different follow-up periods, and differences in laser wavelengths. Pezzi et al. [37], in their case report, described the removal of multiple human papilloma virus lesions with 445 nm diode laser, which resulted in complete tissue healing at 2 weeks after surgery; this finding was different from the present results, probably due to different healing rates of different tissues (mucosal tissue versus fibrotic tissue). Also, it should be noted that their study was a case report, so their results cannot be reliably generalized to all cases.

The current study aimed to compare the use of blue and infrared diode lasers with a surgical scalpel in terms of the complications during and after frenectomy procedures. Due to limited existing data on this subject, our results can serve as a reference for clinicians and researchers in choosing the most effective technique to reduce complications associated with frenectomy procedures. Additionally, the data obtained can assist researchers in developing further studies to confirm the findings of this research. The findings showed the 445 nm blue diode laser’s superiority in terms of bleeding, pain, discomfort while speaking and chewing, and tissue healing, indicating its usefulness and potential advantages over the 980 nm diode laser.

This study had some limitations. Blinding of the participants was not possible. Also, a few patients did not attend the follow-ups, which slightly reduced the final sample size. So, future studies with a larger sample size and inclusion of patients under 18 years of age are recommended. Also, the 445 nm diode laser should be compared with other lasers, such as CO_2_ and Nd: YAG lasers, in oral soft tissue surgical procedures. Due to the limitations of our laser device, the fiber diameters of laser groups were not exactly the same, so matching the fiber diameters should be considered in future studies. Our study utilized continuous mode of laser irradiation. It is important to evaluate the effectiveness of pulsed versus and continuous irradiation. Furthermore, we prescribed chlorhexidine mouthwash following frenectomy, since it is considered as the gold standard for oral cavity antiseptic treatment [38]. However, recent research has linked the use of chlorhexidine to potential side effects like delayed healing [38, 39], Therefore, it may be advisable for future studies to explore alternative topical medications.

## Conclusion

In conclusion, within the limitations of the present study, the results showed that frenectomy with diode lasers was associated with significantly lower intraoperative bleeding, postoperative pain, discomfort in chewing and speaking, and faster healing compared with scalpel surgery. Also, 445 nm blue diode laser was optimal for frenectomy and superior to 980 nm diode laser in terms of the lower level of postoperative pain and faster tissue healing.

### Electronic supplementary material

Below is the link to the electronic supplementary material.

Supplementary Material 1

## Data Availability

All of the data is provided within the manuscript, except for the pairwise and total differences between the three groups, which are shown in supplementary file S1.
