# Supplementary material for: Maxillary labial frenectomy: a randomized, controlled comparative study of two blue (445 nm) and infrared (980 nm) diode lasers versus surgical scalpel
Source: BMC Oral Health. 2024 Jul 25;24:843. doi: 10.1186/s12903-024-04364-w (PMC11271033; doi:10.1186/s12903-024-04364-w)
Supplement: Supplementary file 1 — Supplementary Material 1 [file 12903_2024_4364_MOESM1_ESM.docx]

**Supplementary Table 1**. The pairwise and total differences between the three groups

| Quantitative variable | Pairwise comparison P-value | | | Total comparison (among all three groups) P-value |
| --- | --- | --- | --- | --- |
|  | **Scalpel surgery - 445 nm diode laser** | **Scalpel surgery - 980 nm diode laser** | **445 nm diode laser - 980 nm diode laser** |  |
| Intraoperative bleeding | <0.001 | <0.001 | 0.207 | <0.001 |
| Discomfort in chewing and speaking immediately after surgery | <0.001 | <0.001 | 0.489 | <0.001 |
| Discomfort in chewing and speaking after 7 days | 0.012 | 0.004 | 0.742 | 0.007 |
| Discomfort in chewing and speaking after 30 days | 0.514 | 0.106 | 0.307 | 0.249 |
| Pain score immediately after surgery | <0.001 | 0.589 | <0.001 | <0.001 |
| Pain score after 7 days | 0.009 | 0.279 | 0.001 | 0.002 |
| Pain score after 30 days | 0.405 | 0.229 | 0.065 | 0.157 |
| Tissue healing after 1 day | 0.396 | 0.509 | 0.139 | 0.334 |
| Tissue healing after 7 days | <0.001 | 0.038 | 0.003 | <0.001 |
| Tissue healing after 30 days | 0.178 | 0.289 | 0.024 | 0.074 |
